# Psychological Therapies and the Bipolar Disorder ‘Iceberg’: Going Deeper Than Current Mood and Relapse Prevention Planning

**DOI:** 10.1111/bdi.70099

**Published:** 2026-03-07

**Authors:** Thomas Richardson, Emma Morton, Jennifer Levin, Lauren M. Weinstock

**Affiliations:** ^1^ Southampton Psychosis and Bipolar Research and Innovation Group, School of Psychology University of Southampton Southampton, Hampshire UK; ^2^ School of Psychological Sciences Monash University Clayton Victoria Australia; ^3^ Department of Psychiatry University Hospitals Cleveland Medical Center and Case Western Reserve University School of Medicine Cleveland Ohio USA; ^4^ Department of Psychiatry and Human Behavior Alpert Medical School of Brown University Providence Rhode Island USA

**Key Message**

Psychological therapies for bipolar disorder can operate at different levels. While addressing ‘surface‐level’ issues such as current mood episodes, understanding of bipolar disorder and relapse prevention is key, therapies would be enhanced if ‘under the surface’ issues such as psychiatric comorbidities, childhood trauma and perfectionism are also incorporated.

**Learning points**

- There is fairly strong evidence for psychological therapies reducing acute mood symptoms and reducing the risk of relapse in bipolar disorder.
- There is less clear evidence about psychological therapies to address comorbidities and underlying psychological mechanisms, but these should nonetheless be considered as targets for therapy.

Bipolar disorder (BD) is linked to high levels of disability, significantly impacting functioning and quality of life. In recent years, there has been increasing research supporting the efficacy of psychological therapies, together with medication, to ameliorate mood symptoms and reduce relapse risk. Aside from these two core treatment targets, there are often psychiatric comorbidities and underlying psychological vulnerabilities, which are not addressed by currently available therapies for BD. As a result, such underlying issues are neither assessed nor addressed in terms of research or clinical practice.

This clinical care article outlines our collective clinical, lived experience and academic views on the underlying or ‘deeper’ issues common to individuals with BD, why these should be addressed, and how. An iceberg metaphor is used throughout to illustrate this concept: Some ‘surface level’ issues are the most obvious and clearly visible, but there are remaining difficulties often hidden ‘under the surface’ which nonetheless ‘support’ (or serve to maintain) the current difficulties (see Figure 1). We do not use the term ‘surface level’ in a disparaging way and emphasise our simple hope to encourage clinicians and academics to think ‘deeper’ than these presenting problems.

**FIGURE 1:**
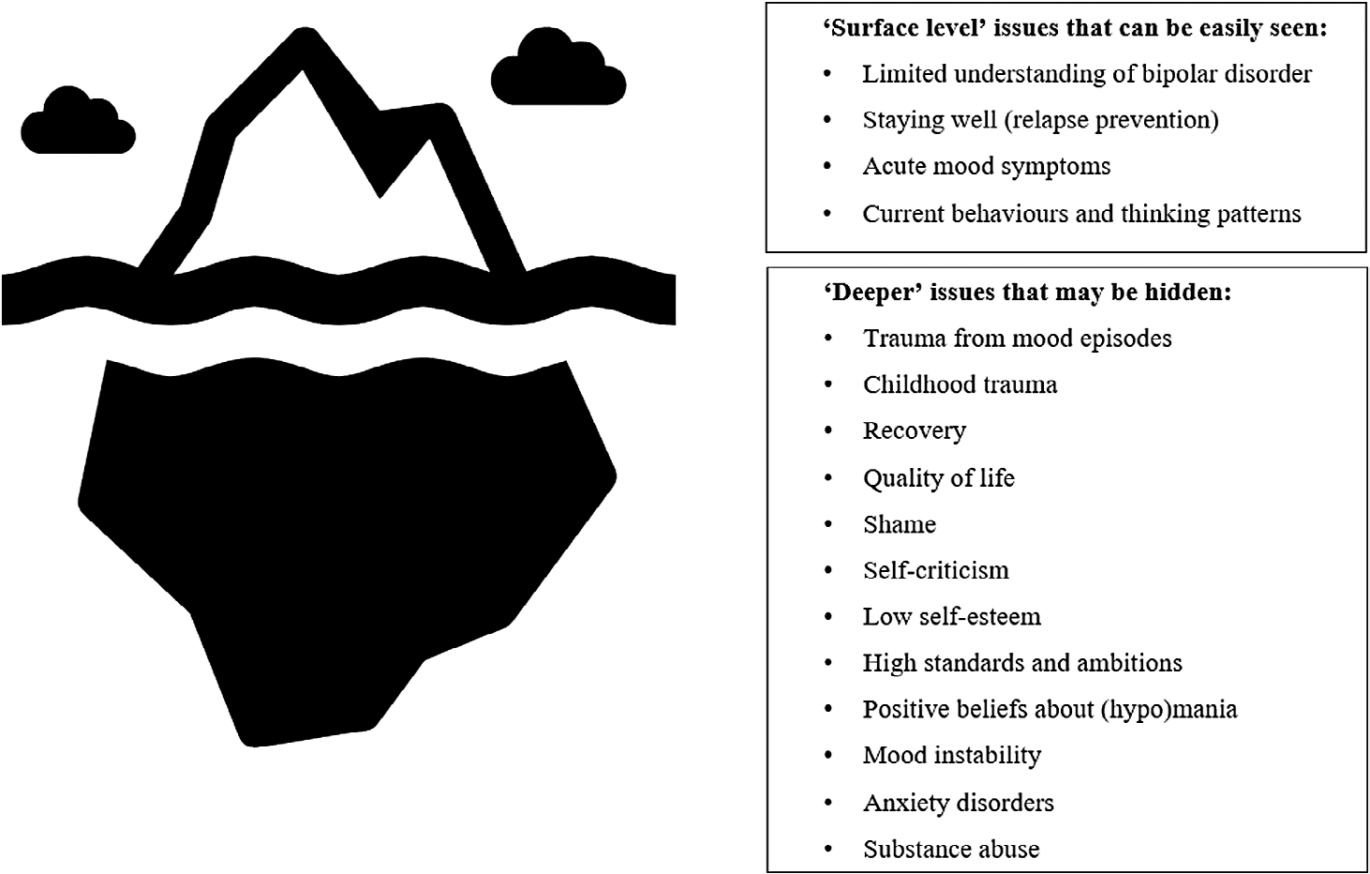
An illustration of the psychological therapies for bipolar disorder iceberg metaphor.

## 1 Psychological Therapies at the ‘Surface Level’

In using the term ‘surface level’ we are referring to the issues that are described as pressing concerns for service users with BD, or immediately visible to clinicians, such as acute symptoms of depression or (hypo)mania. This term also refers to work which may be prioritised due to a stronger evidence base, or restrictions such as session limits.

Guideline‐recommended psychological interventions for BD include Cognitive Behavioural Therapy (CBT), Interpersonal and social rhythm therapy (IPSRT), group‐based psychoeducation, and family‐focused therapies. Although there are individual nuances, these interventions share a focus on reducing current mood symptoms; the therapist will often focus on current thinking patterns (e.g., self‐critical thoughts) and behaviours (e.g., excessive sleeping, withdrawal) which are maintaining the mood episode. Another common presenting concern addressed by current evidence‐based interventions is relapse prevention (also referred to as staying well), through strategies such as understanding triggers for an episode, identifying early warning signs and symptoms, finding behavioural strategies to reduce risk of relapse and developing a shared relapse prevention plan. These treatments address misconceptions, anxieties and questions that the service user may wish to discuss such as ‘Why do I have bipolar disorder?’ and ‘Is there anything I can do besides take medication to stop getting manic?’. As such, these interventions collectively prioritise psychoeducation. Namely, sharing reliable information on the disorder, its triggers and treatments to support acceptance of the diagnosis, medication adherence and engagement in self‐management strategies.

### 1.1 Mood and Relapse Prevention: The Evidence

These ‘surface level’ issues of current mood and relapse prevention are often a key focus in therapy given their strong evidence base. The largest meta‐analysis to date of 39 randomised controlled trials showed that psychological therapies, when used in conjunction with medication, reduce the risk of relapse by 44% [1]. Psychoeducation delivered in a group or family format was shown to reduce risk of relapse compared to individual therapy and CBT, family therapy and interpersonal therapy were found to reduce acute symptoms of depression [1]. Further, the review identified specific skills of cognitive restructuring (a core component of CBT), communication training and regulating daily rhythms which appeared to improve depression symptom severity [1]. The evidence is less clear for (hypo)mania, and therapy conducted during an acute manic episode is challenging, but nonetheless the review also found that cognitive restructuring was linked to manic symptom stabilisation [1]. Thus, there is clear and robust evidence that psychological therapies can improve these ‘surface’ level issues of current mood episodes (and linked thinking patterns), as well as reducing relapse risk.

## 2 Psychological Therapies at a ‘Deeper’ Level

In our clinical experience, as therapy progresses, the service user and clinician will often identify additional difficulties and issues which contribute to the ‘surface level’ presenting problems. These are often less visible or ‘hidden’ when compared to more acute concerns such as preventing another relapse or stabilising mood. Nonetheless, in a similar way to an iceberg where the hidden component underlies and ‘supports’ that which can be seen on the surface, these underlying issues are interlinked and when not addressed, maintain acute mood episodes and trigger relapses (as illustrated by Figure 1). An example may be that a client wants to work on preventing a manic relapse in the future; however, with time it becomes clear through therapy and the collaborative development of a psychological formulation that two driving factors for past relapses are (1) the belief that they are fun and productive when manic, and (2) underlying perfectionism and high standards which drive excessive goal‐focused activity and destabilises mood. Thus, developing a relapse prevention plan will only go so far unless these underlying issues are tackled.

### 2.1 Comorbidities, Underlying Issues and Client‐Valued Goals

Psychiatric comorbidities may represent difficulties which may be less obvious in therapy and may ‘fuel’ current mood difficulties. A recent systematic review [2] found that 38.9% of those with BD have another psychiatric diagnosis, with rates significantly higher than in the general population. Most common comorbidities are anxiety disorders (40.4%), with high rates for specific anxiety disorders such as Post‐Traumatic Stress Disorder (8.2%), social anxiety (10.6%), Generalised Anxiety Disorder (13.1%) and panic disorder (14.9%). The review also found high levels of comorbid substance use disorder (30.7%), Attention‐Deficit Hyperactivity Disorder (18.6%) and eating disorders (9.3%) [2]. It is thus vital to address comorbidities, especially given the availability of evidence‐based psychotherapies for each respective condition, with targeted approaches (e.g., exposure, safety planning) that could be integrated into existing BD psychotherapies.

In addition to formal psychiatric comorbidities, research has demonstrated a clear psychological profile of BD, which may underpin current mood difficulties. Those with BD have been shown to have high levels of impulsivity, low and variable self‐esteem, elevated perfectionism and dysfunctional attitudes (high standards), low self‐compassion, high levels of childhood abuse and high levels of shame [3]. Use of unhelpful emotional coping strategies such as rumination is common amongst people with BD, as is emotional dysregulation during episodes [3]. In addition, those with BD have positive views about extreme mood elevation and a reluctance to ‘give up’ manic episodes (e.g., enjoying the creativity of (hypo)mania), and a positive bias for personal abilities and ambitions to become famous or wealthy [3].

Finally, client‐valued goals such as personal recovery and quality of life may also be at odds with the emphasis of ‘surface level’ treatment frameworks. For example, relapse prevention/staying well work may not be as a high priority for some service users as compared to restoration of occupational and social functioning or dealing with self‐stigma and a disrupted sense of identity. These issues may both represent vulnerabilities that contribute to ‘surface level’ issues, as well as strengths that may be drawn on to guard against future relapses. This may mean that potential perpetuating or protective factors are missed when therapeutic frameworks centre symptom management alone. Furthermore, integration of service user valued goals may promote therapeutic alliance, thereby laying the groundwork for engagement in treatments that focus on key ‘surface level’ issues.

### 2.2 Evidence

Whilst research has demonstrated the high prevalence of comorbidities, and a clear psychological profile of BD, there is much less evidence examining whether psychological therapies can address these comorbidities and psychological mechanisms which may impact the ‘surface level’ presenting problems. A review of publication trends [4] around anxiety disorders in BD found 310 papers published between 2011 and 2020, but only 25 (8.1%) of these were focused on psychological therapies specifically. As a result, whether psychological therapies can reduce anxiety and anxiety disorder comorbidity in BD is less than clear. A similar pattern is observed for other comorbidities such as substance use, and for psychological mechanisms underlying BD such as perfectionism and positive beliefs about (hypo)mania, where there is little or no research on psychological therapies to target these specific mechanisms.

## 3 Conclusion

Psychological therapies have been shown to be effective for addressing ‘surface level’ issues in BD, namely reducing acute mood symptoms and reducing the risk of future relapse. This work is important and should continue, and there is still work to be done to understand treatment moderators and improving access to these therapies [5].

However, we encourage clinicians to think about the ‘deeper’ issues which underly these presenting problems, even if these are not readily bought into therapy by the service user, or are deemed out of the remit of a service or particular therapy. We encourage academics to focus research on better understanding these underlying mechanisms and developing novel psychological therapies to help reduce the impact of difficulties such as childhood trauma and positive beliefs about (hypo)mania. Doing so may lead to longer term, ‘deeper’ and more sustainable changes for those with BD.

## Data Availability

The authors has nothing to report.
